# Strategies to Reduce Mortality in Adult and Neonatal Candidemia in Developing Countries

**DOI:** 10.3390/jof3030041

**Published:** 2017-07-19

**Authors:** Harsimran Kaur, Arunaloke Chakrabarti

**Affiliations:** Department of Medical Microbiology, Postgraduate Institute of Medical Education and Research (PGIMER), Chandigarh 160012, India; drharsimranpgi@gmail.com

**Keywords:** candidemia, developing countries, strategies, mortality

## Abstract

Candidemia, the commonest invasive fungal infection, is associated with high morbidity and mortality in developing countries, though the exact prevalence is not known due to lack of systematic epidemiological data from those countries. The limited studies report a very high incidence of candidemia and unique epidemiology with a different spectrum of *Candida* species. The recent global emergence of multi-drug resistant *Candida auris* is looming large as an important threat in hospitalized patients of developing countries. While managing candidemia cases in those countries several challenges are faced, which include poor infrastructure; compromised healthcare and infection control practices; misuse and overuse of antibiotics and steroids; lack of awareness in fungal infections; non-availability of advance diagnostic tests and antifungal drugs in many areas; poor compliance to antifungal therapy and stewardship program. Considering the above limitations, innovative strategies are required to reduce mortality due to candidemia in adults and neonates. In the present review, we have unraveled the challenges of candidemia faced by low resource countries and propose a ten part strategy to reduce mortality due candidemia.

## 1. Introduction

Candidemia accounts for the majority of invasive fungal infections. The advances in intensive care, interventional technology, transplantation, and aging population invite opportunistic fungal infections. Despite the current transformational improvement in diagnostics and better management strategies, the incidence of candidemia and the attributable mortality rate (25–60%) remains high. Increased hospital stay and higher cost of management are additional worries [1,2,3]. Data on candidemia are mainly available from developed nations of North America and Western Europe. There is a dearth of epidemiological data from developing countries (Asia, Africa, Latin America), which constitute around 80% of the world’s population. The few available studies from those nations demonstrate a high incidence and unacceptably high mortality rate due to invasive candidiasis (IC), and considerable variation in epidemiology of candidemia when compared to developed nations. The challenges in developing countries may be attributed to compromised healthcare due to deficiency of resources and over-capacity patient load in public sector hospitals, non-availability of advance diagnostics, limited awareness of fungal diseases, and inefficient implementation of guidelines [4,5,6,7]. The management of candidemia is largely based on clinical assessment and empirical therapy due to lack of reliable diagnosis and accurate identification of species. The rampant overuse and misuse of antibiotics and steroids by untrained medical practitioners in developing nations complicates the scenario. The infection control practices are sub-optimal in those countries due to lack of infrastructure, staff training, sanitation, surveillance programs, legislation, accreditation of hospitals, and compliance of healthcare workers to general principles of hygiene. Even the limited availability of clean water and soap at hand-washing sites pose hurdles to maintain hand hygiene compliance [6].

With the aim to improve morbidity and mortality due to candidemia, building awareness and development of competent diagnostic mycology laboratories are the initial steps. Local systematic epidemiological studies are essential to formulate strategies to decrease the burden of candidemia, associated morbidity and mortality. The present review describes ten strategies to decrease morbidity and mortality associated with candidemia in neonates and adults in developing countries.

## 2. Differences in Epidemiology of Candidemia between Developed and Developing Countries

The accurate estimation of burden of candidemia is not possible in developing countries where systematic surveillance studies are not conducted. Population-based accurate data is also scarce from developed nations, as the majority of these studies are hospital-based or target specific patient groups, with the exceptions being France, Australia and Iceland [8]. The incidence of candidemia worldwide ranges from 2-14 cases per 100,000 persons and 6.87 per 1000 ICU patients [9,10]. Table 1 summarizes the limited data available on the incidence of candidemia in developing versus developed countries.

It is estimated that the incidence of candidemia has increased five-fold globally in the last 10 years [3]. The developing countries record a 4–15 times higher rate of candidemia as compared to developed countries [16]. The incidence of candidemia reported in developing countries ranges from 0.026 to 4.2 cases per 1000 admissions (Asia 0.026–4.2; Latin America 1.01–2.63; South Africa 0.28–0.36; UAE 0.77), whereas developed countries report an incidence ranging from 0.03 to 1.87 cases per 1000 admissions (USA 0.03; Europe 0.32–1.19; Australia 2.41; UK 1.87) [3,9,11,13,14,17,23,24,25,27]. The available data from intensive care units (ICUs) of developing countries reported the incidence ranging from 2.2 to 41 cases per 1000 admissions, which is considerably higher than ICUs of developed nations reporting an incidence of 0.24–6.87 cases per 1000 admissions [9,16,17]. The incidence of candidemia in paediatric ICUs is exceptionally high in developing countries (42.7 cases per 1000 admissions) compared to the developed nations (0.043–0.47 cases per 1000 admissions) [9,11,12,17]. The overall crude mortality rate due to candidemia in developed nations is below 50% while developing nations are currently struggling with a rate of >50%. (Table 2)

Non-*albicans Candida* (NAC) species have emerged as etiological agents in the majority of candidemia cases worldwide, with an overall decrease in isolation rate of *C. albicans* from 70% to 50% in developed countries [8] (Table 3).

The rate of *C. albicans* isolation is much lower in developing countries, and in some Asian countries the rate has gone down to <10% [46]. Among NAC species, *C. glabrata* is the most common species in developed nations, while *C. tropicalis* and *C. parapsilosis* have emerged as common species in developing countries [9,47,48,49]. *C. glabrata* has been reported as an important opportunistic pathogen in elderly patients and patients on fluconazole prophylaxis in United States [8]. In Latin America, *C. glabrata* isolates are less frequently resistant to fluconazole (10.6–13.2%) compared to North America (18.0%) [50]. *C. tropicalis* is commonly isolated from patients with neutropenia and haematological malignancy in developed countries. In contrast, the agent is isolated from any patient type in Asian countries. This species is well established in tropical regions of Asia where it is the leading cause of candidemia (41.6%). In Latin America, *C. tropicalis* ranks second after *C. parapsilosis* [26]. A multi-drug resistant superbug, *C. auris* is looming large as an important threat across the globe, and the challenge is much higher in Asian countries, as it had been isolated from 5.2% of candidemia patients in ICUs in India [26,51].

The risk factors and underlying diseases for candidemia are similar in both developed and developing countries. In a recent multi-center study on ICU-acquired candidemia from India, interesting observations were made. The patients of relatively younger age, having less co-morbidities, suffered from candidemia. The infection was acquired much earlier post-ICU admission in that study (median 8 days post-ICU admission compared to 23 days in USA) [26]. Though the exact reason for those observations is not known, early colonization of Indian patients and compromise in healthcare due to over-capacity patient load in public-sector hospitals have been postulated as possible factors [26].

The risk factors for candidemia may either be host-related or health-care related. The most vulnerable hosts for acquiring candidemia are those at extremes of age. The comorbidities for a higher risk of candidemia include acute necrotizing pancreatitis, haematological malignancies, solid organ tumours, neutropenia (<500/mm^3^), deteriorating clinical condition, chronic renal insufficiency and previous candidemia attacks [52,53]. The healthcare associated factors include recent surgery (especially abdominal), solid organ transplantation, haemodialysis, longer ICU stay (≥7 days), mechanical ventilation, use of central vascular catheter, total parenteral nutrition, urinary catheter, glucocorticoids and antimicrobial agents therapy, and chemotherapy [9,54]. The study groups from Australia and United States have also reported duration of fluconazole therapy, previous antifungal therapy and intravenous drug use as significant risk factors for development of candidemia [55,56,57]. Further, multifocal *Candida* colonization with colonization index >0.5 or corrected colonization index >0.4 is considered as a significant risk factor for candidemia [10]. The absence of colonization is a strong negative predictor of the disease. It is observed that the candidemia caused by NAC species has an independent association with the use of central venous catheter, glucocorticoids and presence of candiduria [58]. In developing countries, there is hardly any case-control study to delineate the risk for candidemia. However, all the above risk factors exist in the candidemia patients in developing countries.

### Neonatal Candidemia

The majority of cases of invasive fungal infections in neonatal intensive care units (NICUs) are due to *Candida* species (third most common cause of late onset sepsis in <1500 g) [59,60]. Premature neonates are the most vulnerable population for acquiring invasive fungal infections due to their immature immune system and increased use of invasive procedures. The incidence of IC in preterm neonates varies inversely with gestational age. The *Candida* species colonize neonates either by vertical (maternal) or horizontal (NICU environment) transmission [34,61]. The number of sites colonized is directly proportional to the risk of developing IC [34,61]. *Candida* colonization of skin, gastrointestinal, and respiratory tract occurs in 26.7–62.5% of sick neonates within the first two weeks of life [41]. However, in developing countries the *Candida* colonization may occur much earlier, as in an Indian study >70% of the pre-term neonates were colonized within a week and 38% colonized within 24 h of delivery. Low birth weight (<1500 g) pre-term neonates were colonized at multiple sites with high loads of yeast [62]. The extremely preterm (<28 weeks) and extremely low birth weight (ELBW) babies have multiple co-morbidities and face the challenges of prematurity, infections, haemorrhage, necrotizing enterocolitis (NEC), respiratory distress syndrome or congenital anomalies like patent ductus arteriosus [34,61,63]. Further, studies have identified carbapenem use, total parenteral nutrition, central venous lines use, intubation, congenital malformations, low APGAR score and increased hospital stay as risk factors for neonatal candidemia [64,65]. Modern medicine and surgical techniques have enhanced life expectancy of premature and low birth weight (LBW) babies, but have increased the risk of candidemia [34,60,61].

Infants of less than 1 kg are reported to have IC in 7–20% of the cases, while those with weight >1.5 kg have a rate at <1% [66]. The attributable mortality rate was recorded at 12–20% [66,67]. In addition to the high mortality, neurodevelopmental impairment can occur in up to 57% of survivors who are <1000 grams. According to gestational age, the incidence of fungal infections is 20%, 10–20%, 5–10% and <5% in <25 weeks, 25–26 weeks, 27–28 weeks and >28 weeks, respectively [68]. An Indian study showed a high rate (22.8%) of invasive fungal infections in preterm babies with >1 week stay in NICU [69]. Other similar studies from developing countries have reported higher rates at 7.48–8.1% of neonatal candidemia with mortality at 40% and predominance of NAC species isolation (84–86%) [38,39,40]. *C. parapsilosis* is considered the most common NAC species isolated from babies in NICUs [37]. However, in India, the *C. tropicalis* isolation rate is more than *C. parapsilosis* in those neonates.

## 3. Challenges in Diagnosis of Candidemia in Developing Countries

Early diagnosis of candidemia is associated with reduced morbidity and mortality, hospital stay, cost and drug toxicity [70]. The diagnosis starts from suspecting the infection, but the clinical presentation of candidemia is difficult to distinguish from bacteremia, and can be subtle on several occasions. Multiple advanced techniques have been used to diagnose candidemia in well-established laboratories of developed countries. Automated blood culture remains the most common and widely used practice to diagnose candidemia [71]. Bactec 9240 and Bac/T Alert take around 14–72 h for beeping positive signal based on the culture condition and burden of yeast cells [71,72]. Some *Candida* species, especially *C. glabrata*, being slow growers (>5 days), may be missed by available blood culture systems [70]. Lysis centrifugation may improve the recovery of *Candida* species compared to conventional blood culture systems, but the lysis-centrifugation technique is prone to contamination and not used in the majority of laboratories. A large volume (>5 mL) of samples is recommended for better recovery of *Candida*, but it is difficult to obtain from preterm neonates [71]. Approximately 50% of candidemia cases may yield negative blood culture results [73]. Recently, T2 *Candida* panel has been developed and approved by United States Food and drug Administration (FDA), allowing rapid diagnosis and identification of *Candida* species directly from blood [74]. The procedure has been evaluated in a multi-center study reporting good sensitivity (91.1%), specificity (99.4%) and turnaround time (4.4 ± 1 h) [75]. The T2 *Candida* panel can detect five common *Candida* species, which encompasses 95% of *Candida* clinical isolates. However, the spectrum of *Candida* species causing blood stream infection in developing countries is relatively large, leading to the possible chance of missing a considerable number of isolates [26]. T2 *Candida* panel is not available in developing countries.

The identification techniques for *Candida* species are crucial for optimal antifungal therapy, as susceptibility varies among species of *Candida.* The procedure for identification has evolved from conventional manual biochemical-based methods to automated protein and nucleic acid-based methods. Chromogenic primary isolation media, API 20C AUX, VITEK 2, RapID Yeast Plus, MALDI-TOF MS, PNA-FISH, Quick FISH, multiplex PCR, FilmArrayTM BC identification panel and the xTAGTM fungal analyte-specific reagent assay are some of the automated and commercial identification systems used in developed nations. MALDI-TOF MS has immensely revolutionized the identification of *Candida* species by decreasing the turnaround time to <3 h as compared to 2–7 days by conventional as well as sequence-based techniques [76]. The DNA sequencing by Sanger or next generation sequencing helps in the identification of rare yeast species [77].

The available serological tests include detection of either antibody, antigen or metabolites [78]. The detection of (1,3)-β-d-glucan (BDG), a component of fungal cell wall (except *Mucorales*, *Cryptococcus*) is a promising fungal biomarker for early diagnosis of invasive infection and initiation of pre-emptive antifungal therapy [57,79]. Its utility as a diagnostic tool shows an overall sensitivity, specificity, positive and negative predictive values at 47–93.3%, 81.3–100%, 9–89% and 73–100%, respectively. The major concern about BDG test is the false positivity, which creates a problem when only this test is positive in a clinical situation [80,81]. The high price, labor intensive procedure, and the requirement of a specialized laboratory are other limitations. The test cannot distinguish *Candida* infections from other fungal infections. However, it has a high negative predictive value for ruling out *Candida* infections [57]. The test has prognostic value as well with the fall in values after successful antifungal therapy. Jaijakul et al. attempted to correlate BDG levels with the success of treatment by plotting serial BDG levels of candidemia patients over time. They noticed a negative slope in BDG levels of successfully managed patients (PPV 90%) while a positive slope was found in patients with treatment failure (NPV 90%) [82]. The BDG in diagnosis of candidemia in children is not approved by FDA for making paediatric treatment decisions due to limited data [80,81].

*Candida* mannan and anti-mannan antibody detection have gained greater acceptance in Europe than the United States [80,81]. The sensitivity and specificity of both mannan and antimannan IgG reported at 58–93% and 59–83%, respectively, with combined sensitivity of 83% and specificity of 86%. The sensitivity of the tests is good when *C. albicans*, *C. glabrata* and *C. tropicalis* are the etiological agents for IC [80,81]. The tests have a high negative predictive value of 95% and can be utilized to exclude IC. Commercially available Platelia Candida antibody and antigen assays (Bio-Rad, Raymond Poincaré, Marnes-la-Coquette, France) may diagnose candidemia earlier than blood cultures in haematology and ICU patients [57]. The sensitivity of the tests is high for *C. albicans* (100%) and low for *C. parapsilosis* (40%). The *C. albicans* germ tube antibody (CAGTA) assay, an indirect immunofluorescence assay, detects antibodies against the surface of *C. albicans* germ tubes. The test has a sensitivity of 77–89% and a specificity of 91–100% [83].

Many studies evaluated molecular detection methods (PCR) for the detection of candidemia. The *Candida* DNA detection has good sensitivity (95–100%) and specificity (>90%). However, the test lacks standardization and validation by multi-center studies [80,81]. PCR has advantages over BDG or antigen-antibody assays, due to its potential for species identification and detection of molecular markers for drug resistance simultaneously. In Europe, a whole-blood, multiplex real-time PCR assay (SeptiFast, Roche, South Branchburg, NJ, USA) detecting 19 bacteria and 6 fungi (*C. albicans, C. glabrata, C. parapsilosis, C. tropicalis, C. krusei*, and *Aspergillus fumigatus*) has been investigated in diagnosis of sepsis in patients with neutropenic fever. The sensitivity of the test was 94% with a negative result only in *C. famata* candidemia [80,81]. A multiplex nested PCR for detecting seven *Candida* species was evaluated in critically ill paediatric patients at risk of IC. The results are encouraging due to rapidity and sensitivity of diagnosis compared to blood culture [84]. 

The above advanced tests are not available in the majority of the laboratories of developing countries due to their high cost. The diagnosis of candidemia in most centers still relies upon conventional blood culture techniques possessing a long turnaround time and poor sensitivity. The identification of *Candida* species also takes a long time due to the absence of MALDI and molecular techniques in the majority of laboratories. The conventional methods fail to identify the recent outbreak species, *C. auris* [26]. Therefore, a practical solution needs to be adopted in those countries while planning any strategy to reduce mortality due to candidemia.

Few studies have evaluated the role of a known bacterial sepsis marker, procalcitonin (PCT), in diagnosing candidemia [85]. The low PCT value (<2 ng/mL) may act as a cut-off for candidemia differentiating from bacteraemia with a 92% sensitivity, 93% specificity, and 94% negative and positive predictive values [85]. Similarly, the role of C-reactive protein (CRP) levels has been evaluated. A higher CRP value has been found in bacteraemia, compared to candidemia cases. A combination of both CRP and PCT or PCT cut-off 0.5 ng/mL and presence of mannan antigen can also be used with good sensitivity [85]. A low PCT level in a patient with post-surgery sepsis and having risk factors for fungal infections favors candidemia. Charles et al. reported a cut-off at 5.5 ng/mL, which was associated with a 100% negative predictive value and a 65.2% positive predictive value for *Candida*-related sepsis [86]. Further, PCT is considered superior to CRP, serum amyloid A (SAA) and Interleukin (IL)-6 in predicting candidemia [87]. A combination of CRP (with a cut-off value of 116 mg/L), PCT (with a cut-off of 8.06 ng/mL) and IL-6 (with a cut-off of 186.5 pg/mL) has been shown to increase the sensitivity or specificity for diagnosing *Candida* sepsis [87]. However, the tests required validation in a multi-center study.

Due to the limitation of laboratory diagnostics, various clinical prediction rules are proposed to identify the patients likely to have IC [88]. Some of these prediction rules have been validated but none are universally accepted, as each prediction rule has its own limitations. *Candida* scores seem to be a promising tool for identifying high-risk patients who will clearly benefit from early antifungal therapy without a substantial increase of resistant isolates to antifungals. Colonization Index (CI), Corrected Colonizing Index, *Candida* score include *Candida* colonization at multiple sites for predicting IC [57]. This recommendation has been found to be useful to start pre-emptive therapy in surgical ICUs rather than medical ICUs in developed countries [57]. However, in developing countries, a daily colonization study from multiple sites of all patients in ICUs is impractical due to high cost and poorly staffed laboratories. Further, in a recent study from India, it was observed that 97% patients of ICUs were colonized with *Candida* (A K Baronia, personal communication). The Ostrosky’s rule is easier to implement, but only 10% of patients on whom the rule applied develop IC. In a study, this model reaches a negative predictive value of 97%, but sensitivity is only at 34% [89]. 

## 4. Challenges in Management of Candidemia in Developing Countries

The selection of any agent for the treatment of candidemia should take into account a history of recent antifungal exposure, a history of intolerance to any antifungal agent, the *Candida* species isolated and local susceptibility data, severity of illness, relevant co-morbidities, and evidence of involvement of the central nervous system, cardiac valves, and/or visceral organs. The majority of the guidelines recommend the use of echinocandins over azoles due to echinocandin’s broad-spectrum activity and cidal action, its ability to unmask the *Candida* cell wall helping the immune system to target the organism, its activity against biofilm, limited or no drug to drug interactions, better clinical performance than fluconazole and isavuconazole in clinical trials, and better safety than amphotericin B formulations [80]. The initial therapy with echinocandins is considered to be a significant predictor of survival [80]. Although randomized controlled trials (RCTs) have demonstrated treatment success of echinocandins in 70–75% cases, resistance is noted in *C. glabrata* and *C. parapsilosis* [80,90,91,92,93,94,95]. Azole resistance is noted in *C. glabrata* and *C. krusei* isolates while *C. lusitanae* has reduced susceptibility to amphotericin B. The step-down therapy from echinocandins to azoles (fluconazole or voriconazole) has been recommended once the patient is stable and the isolate is azole-susceptible [80,81]. Early removal of the central line is also recommended in catheter-associated candidemia. In neonates, amphotericin B deoxycholate is recommended as the first choice for candidemia while fluconazole may be an alternative drug in patients without previous fluconazole prophylaxis. Additionally, the dosing in paediatric patients may vary depending on pharmacokinetics of antifungal agents. The safety, efficacy, area under the curve, and maximal concentration (2–5 µg/mL) of amphotericin B lipid complex (ABLC) are similar in adults and children [96]. The volume and clearance of liposomal amphotericin B varies with weight in neonates and children [97]. The pharmacokinetics of azoles is quite variable in children due to rapid clearance in them [80,98,99,100]. Caspofungin and micafungin are approved by the FDA for use in children whose dosing is based on body surface area rather than weight [101]. 

The major challenges in antifungal therapy in developing countries include delayed initiation of therapy due to late diagnosis, use of inappropriate drugs, improper dosing and duration of antifungal treatment. The lack of adequate diagnosis has resulted in overutilization or underutilization of antifungal agents in many instances [54]. Instead of directed therapy after diagnosis, the use of an antifungal drug is decided on the basis of fever-driven approach. If any patient with suspected sepsis does not respond to broad-spectrum antibacterial therapy for 3–5 days, an antifungal is added as empirical therapy. The majority of patients in developing countries are economically deprived and under privileged, who are unable to bear the cost of antifungal agents. Governments supply free medicine in very few countries. The limited availability of antifungal drugs, and the use of generic medicine without thorough accreditation of manufacturing process are other barriers in these countries. Amphotericin B deoxycholate is commonly used despite its high toxicity. The lipid preparations of amphotericin B are not available in the majority of hospitals in Latin America and Asia [102]. Fluconazole is the main drug used in IC even in unstable and neutropenic patients due to cost consideration. Fluconazole is the most frequent antifungal agent used as primary therapy for candidemia in Latin America, despite high mortality rates [50]. In an Indian multi-center study on ICU-acquired candidemia, fluconazole was used as therapy in 64% of patients [26]. Antifungal susceptibility testing is required for the proper choice of antifungal drug. The developing countries still struggle with proper infrastructure for this facility [103]. There is a lack of country-specific guidelines in developing countries to guide the therapy, which forces them to follow the guideline of developed nations though their local epidemiology may be different from them. Few experts in Latin America have made recommendations for the treatment of candidemia in adults, children and neonates based on current clinical evidence, the regional epidemiology, and expert opinions [50,102,104]. Such expert recommendation is also lacking in the majority of the developing countries. Poor infection control practices in those countries add to the impediment while managing any patient with candidemia.

## 5. Strategies to Reduce Mortality and Morbidity Due to Candidemia in Developing Countries

Considering the above challenges, 10 rational steps are proposed to reduce mortality in patients with candidemia in adults and neonates of developing countries.

1. Development of reference laboratory and improvement of mycology laboratories: Small countries require at least one reference laboratory and multiple in large countries. Establishment of more numbers of laboratories is essential to cater to large populations in developing countries. The government is required to spend more money to meet those essential needs.
2. Improvement in diagnosis: Although the resources in developing countries are limited, reasonable alternatives may be implemented. Firstly, maintaining a high level of suspicion particularly in high risk patients may expedite the investigation process. Secondly, better sample collection will improve diagnosis. Thirdly, the identification of positive cultures may be accelerated by collaboration among multiple centers of a region pooling fund for purchase of a MALDI-TOF whose running cost after initial installation is very low and affordable for developing countries. Fourthly, although specific fungal serological markers are expensive, cheaper alternatives like PCT and CRP, which have shown a high negative predictive value may be standardized and validated for excluding candidemia. Fifthly, standardization of *Candida* PCR may prove cost effective in the long run for early diagnosis. Sixthly, a good communication between mycology laboratory and clinicians and critical call alert would help in early antifungal therapy. Emergency laboratories should function around the clock and should be equipped with automated blood culture systems. The results should be communicated to the clinician in a real-time fashion.
3. Education: The importance of diagnosis of candidemia must be included in the curriculum of residents and health care workers. Simple educational programs including lectures, posters, hands on training and self-study modules for physicians and nursing staff will lead to a significant decrease in catheter line-associated blood stream infection (CLABSI) rates. Educational programs along with periodic reassessment of health care worker knowledge regarding infection-prevention practices are necessary for compliance to evidence-based practices.
4. Improvement of infection control: Training in infection control is of paramount importance. Hands of health care workers are the frequent source and transmitters of *Candida* from patient to patient and environment to patient [105]. Adequate maintenance of hand hygiene reduces rates of nosocomial infections and cross-transmission as shown in many studies over the last few decades [106]. Improving hand hygiene and optimal catheter placement help to reduce sepsis episodes. A significant reduction (16.9% to 9.9%) in nosocomial infections was noticed after a hand hygiene promotion program over a period of four years in a developed country [107]. Hand hygiene also reduced CLABSI rate by 72% in patients receiving parenteral nutrition [108]. The use of World Health Organization (WHO)-advocated alcohol-based hand rubs is a practical solution to overcome the problems of hand hygiene in developing countries. Audits are required to estimate the compliance and reason of non-compliance of infection control practices.
5. Source control: Source control is implemented to control a focus of infection and reduce the favorable conditions that promote microorganism growth or that maintain the impairment of host defenses [109]. The removal of any pre-existing central vein catheters or abscesses or other fluid collections will help to reduce mortality due to candidemia [88]. It is recommended to remove the central venous catheters (CVCs) as early as possible in candidemia in non-neutropenic patients when the source is presumed to be the CVC [80]. In the neutropenic patient, the decision of removing CVC is individualized, as the source of *Candida* in this group is generally other than a CVC (e.g., gastrointestinal tract) [80]. Many studies have shown the effect of both timing of therapy and/or source control on mortality. Early initiation of appropriate antifungal therapy and removal of CVC or drainage of infected material are associated with better overall outcomes [57,88,110,111,112,113]. Moreover, mortality in candidemia patients with septic shock reaches 100% if an antifungal is not begun within 24 h and the source is not controlled [80]. Giuliano et al. demonstrated the use of topical prophylaxis with nystatin and adequate CVC management in neurosurgical ICU to prevent IC [114]. Lagunes et al. (Spain) conducted a retrospective, multi-center, cohort study in surgical wards and ICUs and reported adequate source control in 60% of patients with intra-abdominal candidiasis (IAC) within 48 h of diagnosis [109]. They identified inadequate source control as the only common risk factor for 30-day mortality in both ICU and non-ICU groups. Better survival was observed in patients receiving both proper source control and antifungal therapy.
6. Local epidemiology: A wide range of variation is observed in *Candida* species distribution between developing countries, even within the countries [95,115,116]. While the culture/susceptibility data are yet to be released by laboratories, the treatment decisions are based on the knowledge of local epidemiology (frequency of isolation and antifungal susceptibility of each *Candida* species) [80]. Moreover, real time data generation on antifungal susceptibility is a challenge in most of the centers of developing countries. The information maintained by the microbiology laboratory may be circulated regularly to clinicians and antifungal stewardship teams for therapeutic decisions. It will also help in planning local candidemia management strategies. Many regions in United States, Europe and few developing countries conduct both sentinel and population based surveys over many years and keep records. The 2011 WHO rapid advice guidelines for appropriate antifungal regimens may be incorporated into country-specific or region-specific treatment guidelines [117].
7. Prophylaxis: Antifungal prophylaxis in high-risk adults and premature low birth weight neonates is an important strategy aiming at reduction of mortality due to candidemia. Although few studies have shown effectiveness of echinocandin prophylaxis in transplant patients, fluconazole, which is a cheaper alternative, is also beneficial and affordable in developing countries. Weekly fluconazole prophylaxis instead of daily dosing may be cost-effective for haematological or neutropenic patients to decrease morbidity and mortality due to candidemia [118]. However, prophylaxis in adult ICUs (overall rate of IC <5%) is recommended only in selected patient groups [80,119]. A meta-analysis by Cruciani et al. demonstrated a decrease in rate of candidemia (relative risk 0.3), attributable mortality rate (RR 0.25) and an overall mortality rate (RR 0.6) by fluconazole prophylaxis, thereby strengthening the use of fluconazole as a cheap alternative for developing countries [120]. However, the risk benefit ratio is required to be optimized while giving fluconazole prophylaxis especially after emergence of *C. auris* in many developing countries and the rise of fluconazole-resistant *Candida* species infection rate.

Studies in neonatal populations have shown a reduction of *Candida* related mortality by 80–90% and occurrence of IC by 80% after fluconazole prophylaxis. Even nystatin prophylaxis decreases mortality by 50–60% [121,122]. Healy et al. reported elimination of *Candida* related mortality after using fluconazole prophylaxis in <1000 g neonates in NICUs [123,124,125]. The efficacy of fluconazole prophylaxis in extremely preterm infants is shown to be 95%, 85% and 88% in <750 g, 1000 g and <28 weeks, respectively [122]. It is clear that fluconazole prophylaxis is beneficial within two days of birth in preterm infants <1000 g or <28 weeks until central/peripheral access is not required [122].

8. Treatment: Appropriate early management of IC is required to reduce hospital or ICU stay, thereby decreasing the eventual cost of hospitalization and management, and finally reducing mortality due to candidemia. It is generally believed that the early institution of antifungal therapy (fluconazole/echinocandins) within 12–72 h of positive culture prevents mortality (1.5–2 times patients survive) in adults in ICU [110,111,126]. On the contrary, few studies did not find any role of early antifungal therapy in decreasing mortality [127]. It had been shown that high mortality still occurs even when the antifungal is initiated in a timely manner [112]. Lopez-Corter et al. observed no higher mortality in a multi-center study when empiric or target therapy included fluconazole in place of echinocandins, supporting the use of fluconazole safely in developing countries [128]. They even denied the preference of echinocandins in severely ill patients. The antifungal susceptibility data of the developing countries can guide the use of fluconazole as effective antifungals in susceptible isolates. Additionally, amphotericin B deoxycholate can be used as a cost-effective alternative especially in neonates.
9. Availability of antifungal drugs: A big barrier is faced by low-income and middle-income countries to access antifungal agents, and the drug cost [117]. In many developing countries, medicine regulatory authorities are compromised by insufficient resources and human capacity. Drug companies should consider low pricing for developing countries, which may be ensured by providing incentives to pharmaceutical manufacturers for producing generic versions of the drugs. Currently, amphotericin B is not available in 42 developing countries and lacks license in 22 countries. In the process, around 6.6% of the global population do not have access to amphotericin B [129].

Efforts should be made to include these drugs to the WHO core Essential Medicines List (EML) and register them in low-income and middle-income countries. Of the antifungals used for managing candidemia, amphotericin B and fluconazole are on the WHO EML. The available data shows a listing of amphotericin B on the EML in 11 countries only [129]. Although fluconazole was listed as an Essential Drug by the WHO as early as 1999, it is still not included in country essential medicine list in many developing countries like Colombia, Gabon, Poland, Serbia, Lebanon, Indonesia, Malaysia, Bhutan and Surinam [129].

10. Antifungal stewardship: The rational use of antifungal agents in health care institutions must be followed for monitoring and guiding the appropriate antifungal use including dosing, duration of therapy, and route of administration [130]. The aim of this program is to achieve the best outcome without unnecessary adverse reactions and emergence of drug resistance [54]. Apisarnthanarak et al. from Thailand have shown the success of a program comprising of education, antifungal hepatic and/or renal dose adjustment chart, specific prescription forms for antifungal drugs and prescription-control approach [130,131]. They noticed a 59% reduction in antifungal prescriptions, a significant decrease in inappropriate antifungal use (71% to 24%), continuous overall reduction in antifungal use and significantly lower fluconazole use. For the success of the program, an efficient teamwork and adequate hygiene and standard precautions are necessary which must be monitored regularly by infection control nurses. However, the programs in developing countries generally depend on individual efforts of infectious disease physicians rather than teamwork [132].

One way of managing the misuse or overuse of antifungals in developing countries is by making prior approval from an infectious disease physician mandatory before use. De-escalation should be planned after microbiology reports. Transition from parenteral to oral therapy can further lower the hospital stay and cost. The pharmacoepidemiologic data should be monitored in developing countries for evaluating the prescribing trends and identifying areas for improvement and correlate with emerging drug resistance. A periodic survey of epidemiological data should be performed in neonatal areas as well, including speciation and antifungal susceptibility testing of *Candida* isolates [34]. 

Finally, the national health authorities of developing nations should promote antifungal stewardship programs by evaluating the true scenario of antifungal consumption and strengthening the infection control procedures**.**

## 6. Strategies Specific for Neonates

- Antifungal prophylaxis*:* Antifungal prophylaxis as discussed previously is most beneficial for preterm infants <1000 grams and/or ≤28 weeks’ gestation from birth until they no longer require central/peripheral access.
- Maternal vaginal candidiasis screening and decolonization: Preterm infants are colonized by *Candida* from maternal flora [133]. Studies from developing countries have reported a prevalence of vaginal candidiasis ranging from 14.6% to 42.9% in pregnant females [134,135]. Screening and management of maternal vaginal colonization and candidiasis may help prevent neonatal colonization at an early stage. Even empiric therapy for antepartum women has been suggested [34].
- Neonatal medication restriction: The use of broad-spectrum antibiotics, especially third and fourth generation cephalosporins and carbapenems, acid inhibitors and steroids in preterm babies is linked to an increased risk of *Candida* infection [133]. The usage of an aminoglycoside instead of cephalosporin or carbapenem as an empiric therapy may reduce the risk of IC. Moderate evidence exists for restricting the use of H2 blockers and PPI in gastritis. Similarly, the use of dexamethasone in intubated infants is associated with increased risks of IC and candidemia (10%), respectively [133].
- Early breastfeeding and enteral feeding: Necrotizing enterocolitis (NEC) is reported to be associated with high rates of fungal infections (16.5%) [133]. The early establishment of breastfeeding within 3 days of life has shown decreased rates of fungal infections in infants of <1000 g due to development of a favorable microflora in the neonate. Early enteral feeding also promotes the development of healthy gut microflora [34]. The studies regarding the effect of risk factor reduction need to be evaluated in neonates in developing countries.
- Lactoferrin and probiotic administration: Bovine lactoferrin alone or in combination with probiotics given to <1500 g of neonates in an RCT showed a decreased incidence of late onset sepsis although the sample size of the trial was small [34]. Clinical trials conducted in preterm neonates demonstrated a favorable effect of *Saccharomyces boulardii* containing probiotics without any evidence of fungemia or sepsis [136]. However, few studies reported occasional cases of fungemia subsequent to the use of probiotics, questioning the safety of these products [136,137,138,139,140]. It is therefore recommended to use probiotics cautiously in pre-term neonates and immunocompromised patients [139].
- Heightened infection control: Chen et al. reported implementation of aggressive hand hygiene practices in addition to fluconazole prophylaxis to be more successful in preventing candidemia in preterm infants of <33 weeks in NICU than prophylaxis alone [13]. Chitnis et al. further demonstrated a significant reduction (75%) in the overall incidence of candidemia in NICUs due to improved central line insertion and maintenance practices over a period of 10 years [141]. These data suggest the successful contribution of heightened infection control in NICUs.

## 7. Future Perspectives

Rapid diagnostic methods, which are cost-effective with good sensitivity and specificity, need to be standardized and validated for use in developing countries. Development of newer antifungals in the future may overcome the menace of drug resistance, and decrease mortality. The early vaccine trials for a safe and effective vaccine targeting high risk patients (especially IC patients) may improve the scenario further [142]. Currently, two vaccine candidates in clinical trials, namely NDV-3 and rHyr1p-N, are prophylactic recombinant vaccines with an Alhydrogel^®^ adjuvant against the N-terminal region of the agglutinin-like sequence 3 protein (Als3p) and Hyr1p, respectively [142]. 

## Figures and Tables

**Table 1:** Country-wise incidence of candidemia.

Population based (n/100,000 population) (Developed countries)					
Country	Incidence	Reference	Country	Incidence	Reference
North America	13.3 to 26.2 (9.4–75 neonates; 5.2–26 elderly)	[9]	Norway	24	[9]
USA	3.65–26.2	[9,11,12]	Sweden	4.2	[9]
Australia	1.81–2.41	[9,13]	Spain	4.3–8.1	[9]
Europe	9.4	[9,14]	Iceland	5.7	[15]
Denmark	8.6–9.4	[9]	Canada	2.8	[9]
Finland	1.9–2.86	[9]	England and Wales	1.52 (infants 11)	[9]
**Hospital based data (^a^, per 1000 admissions; ^d^, per 1000 discharges; ^pd^, per 1000 patient days; ^py^, per 1000 patient years)**					
**Developing Country**	**Incidence**	**Reference**	**Developed Country**	**Incidence**	**Reference**
Overall Asia	^a^ 0.39–14.2 ^pd^ 0.026–4.2	[16,17]	USA	^d^ 1.9–2.4 ^a^ 0.30 ^pd^ 0.46	[11,12]
Korea	^py^ 29	[16]	Canada	^a^ 0.45	[9]
China	^pd^ 0.026–0.05 ^a^ 0.32–0.55	[16,17,18,19]	UK	^bd^ 0.109 ^pd^ 0.03 ^a^ 1.87	[9]
Hong Kong	^pd^ 0.07 ^d^ 0.25	[16,17]	Australia	^a^ 0.21	[13]
Taiwan	^d^ 1.2–2.93 ^pd^ 0.14–2.8	[16,17]	Switzerland	0.049	[9]
India	^a^ 1–12 ^d^ 1.94 ^pd^ 1.24	[8,16,17]	Sweden	^a^ 0.32 ^pd^ 0.44	[9]
Thailand	^a^ 1.32 ^d^ 1.31 ^pd^ 0.12–0.15	[16,17]	Belgium	^a^ 0.44 ^pd^ 0.065	[20]
Turkey	^a^ 0.56–5.1 ^pd^ 0.058–0.30 ^d^ 0.42	[9,16,21]	France	^a^ 0.2–3.8	[22]
Singapore	^pd^ 0.12–0.33	[16,17]	Spain	^pd^ 0.073–0.136	[9]
Japan	^pd^ 0.0004–0.0008	[9]	Italy	^a^ 0.38–1.19 ^pd^ 0.12–0.31	[9]
South Africa	^a^ 0.28–0.36	[23]			
Latin America	^a^ 1.01–2.63 ^pd^ 0.23	[9,24]			
Argentina	^a^ 1.95 ^pd^ 0.24	[9]			
Venezuela	^a^ 1.72	[9]			
Brazil	^a^ 1.38–2.49 ^pd^ 0.26–0.37	[9]			
Honduras	^a^ 0.90 ^pd^ 2.5	[9]			
Ecuador	^a^ 0.90 ^pd^ 0.16	[9]			
Chile	^a^ 0.33 ^pd^ 0.09	[9]			
Columbia	^a^ 1.96 ^pd^ 0.16	[9]			
UAE	^d^ 0.77	[25]			
**Special groups (^a^, per 1000 admissions; ^d^, per 1000 discharges; ^pd^, per 1000 patient days; ^py^, per 1000 patient years)**					
**Developing Country**	**Incidence**	**Reference**	**Developed Country**	**Incidence**	**Reference**
Overall Asia; ICU	^a^ 2.2–41 ^pd^ 2.2 ^a^ 42.7 NICU/PICU)	[17,26]	Europe; ICU	^a^ 2.6–16.5 ^pd^ 0.07–0.33	[9]
China; ICU	^a^ 3.2	[9]	EPIC II study; ICU	^a^ 6.87	[14]
India; ICU	^a^ 6.51	[26]	Germany; ICU	^a^ 0.24 ^pd^ 0.07	[9]
Turkey; ICU	^a^ 12.3–42.7 ^pd^ 2.31	[9,26]	France; ICU	^a^ 6.7	[9]
Korea; ICU	^d^ 9.1	[16]	Italy; ICU	^a^ 0.26–1.65 ^pd^ 0.33	[9]
Hong Kong; ICU	^pd^ 2.2	[16,17]	US; Haematological malignancy	^pd^ 0.19	[9]

ICU = Intensive Care Unit; NICU = Neonatal Intensive Care Unit; PICU = Paediatric Intensive Care Unit.

**Table 2:** Country-wise mortality rates due to candidemia.

Developing Countries	Crude Mortality Rate	References	Developed Countries	Crude Mortality Rate	References
China	28.1–36.9% ^n^ 15.7% ^n^ 8.9% (ICU)	[18,28,29,30]	USA	19.6–40% ^p^ 13% ^elbw^ 37% ^n^ 12–19%	[31,32,33,34]
Japan	26%	[35]	Switzerland	44–46%	[31,36]
India	^n^ 34.9–40%	[37,38,39,40]	Spain	44–47%	[31,36]
Pakistan	^i^ 24–75%	[41]	Canada	30–52%	[31,36]
Taiwan	36.7–59%	[17]	Italy	35%	[31,36]
Kuwait	15–60%	[42]	Australia	21% (SOT)	[43]
Brazil	50–72.2%	[44]	USA	26.5% (SOT)	[45]
South Africa	60%	[23]			

^n^, neonates; ^i^, infants; ^p^, paediatrics; ^elbw^, extremely low birth weight; ICU, intensive care unit; SOT, solid organ transplant.

**Table 3:** Country-wise distribution of *Candida* species causing candidemia [9,26].

Country	*C. albicans* (%)	*C. tropicalis* (%)	*C. parapsilosis* (%)	*C. glabrata* (%)	*C. krusei* (%)
Developing Countries					
Latin America	43.6–51.8	13.2–17	10.3–25.6	5.2–7.4	1.4
Argentina	38.4–42.5	15.4–16.8	23.9–26	4.3–6.2	0.4–1.8
Brazil	40.5	13.2	25.8	10	4.7
Chile	42.1	10.5	28.9	7.9	7.9
Columbia	36.7	17.4	38.5	4.6	-
Ecuador	52.2	10.9	30.4	4.3	-
Honduras	27.4	26.7	14.1	3.7	3
Asia Pacific	56.9–64.4	11.7	7.4–13.7	12.6–13.7	1.2–2
China	35.9–41.8	17.6–21.8	7.7–23.8	12.3–12.9	-
India	20.9	41.6	10.9	-	-
Thailand	35.6	27.1	15.7	16.3	-
Turkey	45.8	24.1	14.5	4.8	-
Africa and Middle East	67.1	6.6	6	8.8	1.6
South Africa	45.9	3.3	25	19.8	3.3
**Developed Countries**					
USA	38–48.9	7.3–10.5	13.6–17.1	21.1–29	1.9–3.4
Canada	64	11	11	11	-
Europe	55.2–67.9	4.9–7.3	4.2–13.3	11.3–15.7	1.9–3.4
Belgium	55	2.8	13	22	2.3
Finland	67–70	2–3	5	9–19	3–8
Germany	58.5–66	7.5	8	19.1	1.4
Italy	40.2–50.4	8.2–9.8	14.8–36.9	9.8–20.3	-
Norway	69.8	6.7	5.8	13.2	1.6
Spain	36.5–49	5.9–10.7	20.7–46.8	3.9–13.6	1–2.1
Sweden	60.8	2	8.9	20.1	1.2
Switzerland	68	9	1	15	2
UK and Wales	53.7–64.7	3.2–4.4	7.4–10.7	16.2–25.8	1–2.9
Australia	44.8	4.8	16.5	26.7	2.6
